# Development and validation of a patient‐reported outcome measure for patients with chronic respiratory failure: The CRF‐PROM scale

**DOI:** 10.1111/hex.13324

**Published:** 2021-08-01

**Authors:** Hangzhi He, Hao Li, Xianhua Zeng, Hui Zhao, Yanbo Zhang

**Affiliations:** ^1^ Department of Health Statistics Shanxi Medical University Taiyuan Shanxi Province China; ^2^ Respiratory Medicine The Second Hospital of Shanxi Medical University Taiyuan Shanxi Province China

**Keywords:** chronic respiratory failure, classical test theory, item response theory, patient‐reported outcome

## Abstract

**Background:**

Various health‐related quality‐of‐life (HRQOL) tools are used to evaluate patients with chronic respiratory failure (CRF), but there is a relative lack of tools available for the evaluation of social support and treatment in these patients. The present study focused on the development of a systematic patient‐reported outcome measure (PROM) tool for use in patients with CRF.

**Methods:**

The CRF‐PROM scale conceptual framework and item bank were generated after reviewing the corresponding literature and HRQOL scales, interviewing CRF patients and focus groups. After creation of the initial scale, the items in the scale were selected through two item selection theories, and the final scale was created. The reliability, validity and feasibility of the final scale were assessed.

**Results:**

The CRF‐PROM scale includes four domains (i.e., physiological domain, psychological domain, social domain and therapeutic domain) and 10 dimensions. After the item selection process, the final scale included 50 items. Cronbach's *α* coefficients, which were all above 0.7, indicated the reliability of the scale. The results of structural validity met the relevant standards of confirmatory factor analysis. The response rates of the preinvestigation and the formal investigation were 93.3% and 97.6%, respectively.

**Conclusions:**

The CRF‐PROM scale developed in the present study is effective and reliable. It could be used widely in the posthospital management of patients, in CRF studies and in clinical trials of new medical products and interventions.

**Patient or Public Contribution:**

Participants from eight different hospitals and communities participated in the development or validation phase of the CRF‐PROM scale.

## 1 INTRODUCTION

Chronic respiratory failure (CRF) occurs in the advanced stages of many respiratory diseases, and it is associated with a high hospitalization rate and high mortality.^1^ It continues to affect the quality of life of patients posthospitalization^2^ in terms of physical and psychological factors as well as factors associated with social support, satisfaction with treatment and treatment compliance, among others.

In previous reports, it was proposed that the assessment of the effects of treatment on any individual patient should include the patient's own evaluation of therapy, or patient‐reported outcome.^3^, ^4^ A patient‐reported outcome measure (PROM) is any report of the status of a patient's health condition that comes directly from the patient, without interpretation of the patient's response by a clinician or anyone else.^5^, ^6^, ^7^ The importance of the assessment of quality of life to evaluate the human and financial costs and benefits of modern medical techniques has become increasingly recognized in research and healthcare practice.

Currently, there are many scales for estimating quality of life, but there is no scale specifically designed for use in patients with CRF. When it is necessary to estimate quality of life in patients with CRF, a universal scale (such as SF‐36^8^) or a chronic obstructive pulmonary disease (COPD) scale is usually used.^9^ Generic instruments are useful for comparing effects on quality of life in populations with different diseases; however, disease‐specific tools are generally more sensitive to disease‐specific issues and are therefore more appropriate for clinical trials in which specific therapeutic interventions are being evaluated.^10^, ^11^ Previous research in patients with CRF has mainly focused on their physical condition and athletic ability.^12^ Few studies have investigated changes in psychological state caused by disease, or factors such as social support, treatment compliance and therapeutic satisfaction.

The aim of the present study was to develop a new PROM scale for use in patients with CRF that was comprehensive and showed sufficient validity, reliability and feasibility. The intention was to develop a scale that was a useful tool for posthospital management and in clinical trials.

## 2 METHODS

### 2.1 Ethics approval and consent to participate

The study and the CRF‐PROM were reviewed and approved by the Medical Ethics Committee of Shanxi Medical University, China (No. 2018LL128), and written informed consent was obtained from all participants.

### 2.2 Study population

Participants were enrolled from eight different hospitals and communities in Shanxi Province, China. The scale was usually filled out by participants independently, but in cases where participants were not able to do it unassisted, the questions were asked verbally by a trained investigator. The inclusion criteria were age 18 years or older and willing to participate in the study. The CRF group included patients diagnosed with CRF by a clinician and the control group included healthy subjects from the communities mentioned above without respiratory failure or malignant tumour of the respiratory system. The exclusion criteria were mental illness or presence of a consciousness disorder and inability to understand or complete the scale for any reason.

### 2.3 Development of the CRF‐PROM

**Figure 1:**
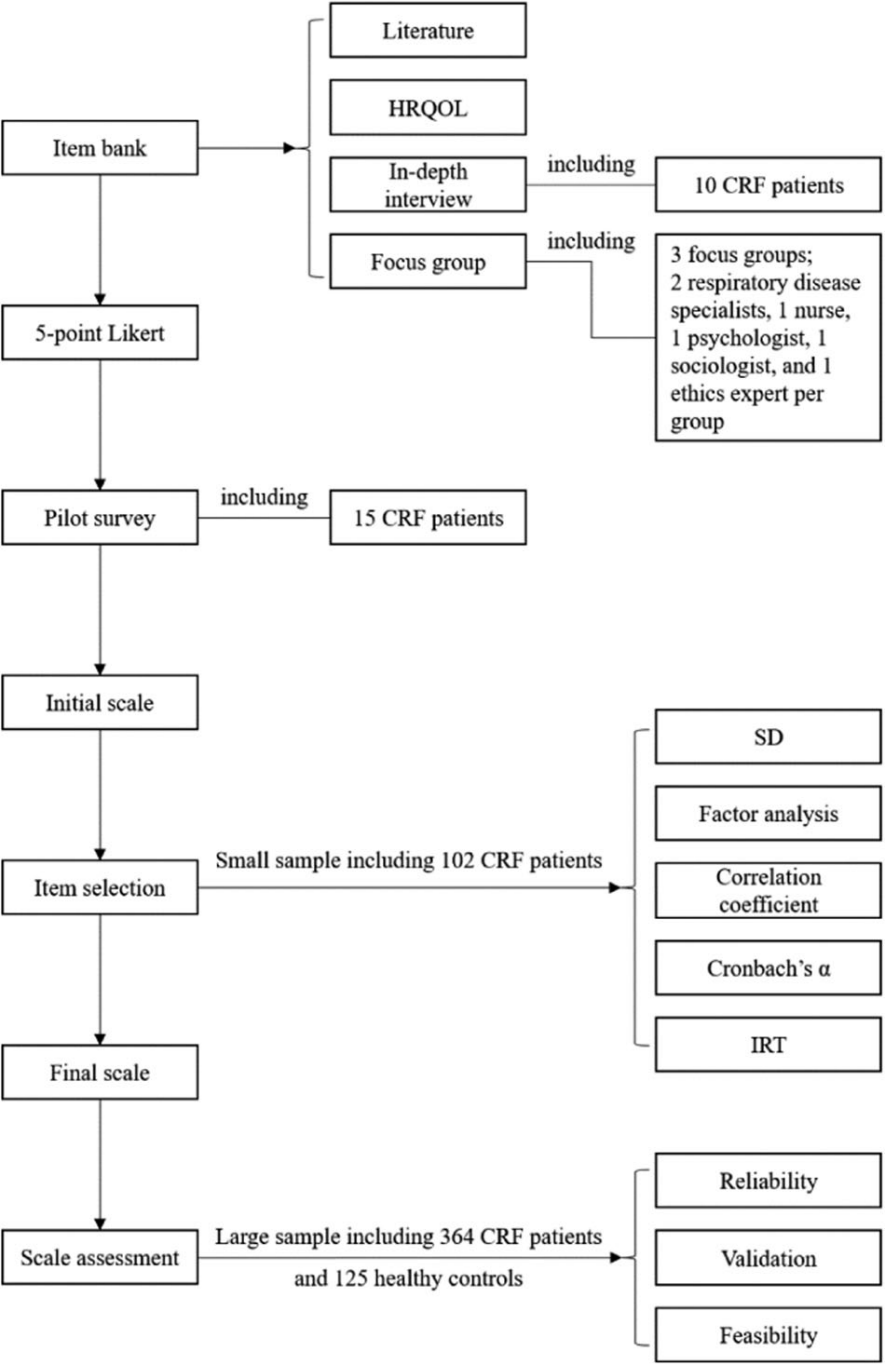
Flowchart of the CRF‐PROM developmental process. CRF, chronic respiratory failure; HRQOL, health‐related quality of life; IRT, item response theory; PROM, patient‐reported outcome measure; SD, standard deviation

The CRF‐PROM was developed in four phases: (1) creation of an item bank, (2) development of the initial scale, (3) selection of items and (4) scale validation. A flowchart of the developmental process is shown in Figure 1.

#### 2.3.1 Creating an item bank

The present study was conducted in strict accordance with the principles and procedures for the production of scales as defined by the US Food and Drug Administration (FDA). Based on patients' own feelings, a scale for evaluating clinical outcomes in patients with CRF was developed. The scale is a multidimensional assessment. In accordance with the purpose and criteria of being 'patient‐centred', the theoretical framework of a PROM scale should incorporate physiological, psychological, social and therapeutic components. The scale developed is a patient self‐assessment.

**Figure 2:**
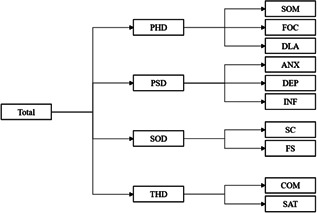
Conceptual framework of the CRF‐PROM. ANX, anxiety; COM, compliance; CRF, chronic respiratory failure; DEP, depression; DLA, daily living activity; FOC, function of cognitive; FS, family support; INF, inferiority; PHD, physiological domain; PROM, patient‐reported outcome measure; PSD, psychological domain; SAT, satisfaction; SC, social contacts; SOD, social domain; SOM, somatization; THD, therapeutic domain

Based on extensive literature consultation and other health‐related quality‐of‐life (HRQOL) scales, a theoretical model of the respiratory failure scale was established (Figure 2). In this stage, 10 patients (male/female ratio: 1.5; average age: 68.3 years) were selected for one‐on‐one interviews, and three focus groups were organized. Each focus group included two respiratory disease specialists, one nurse, one psychologist, one sociologist and one ethics expert. They generated specific suggestions for revision of the scale in various domains together. The duration of each of the aforementioned one‐on‐one interviews with 10 patients was no less than 30 min, and the impact of disease on their quality of life was documented. An item bank was then created.

#### 2.3.2 Formation of the initial scale

The items in the questionnaire are all based on a 5‐point Likert scale, and it includes positive items (with higher scores corresponding to better quality of life) and negative items (with higher scores corresponding to lower quality of life). The score range is 0–4, and scores pertaining to negative items are converted into negative numbers when calculating the total scores.

We selected 15 patients for a pilot survey to ascertain whether all items were accurately understood. Each patient completed the scale independently, and then explained their interpretation of every item to the investigator. Any misunderstood items were modified.

#### 2.3.3 Item selection

To ensure that the final scale has good reliability, validity and feasibility, the selection process followed the principles of good sensitivity, independence, representativeness and internal consistency. We utilized both the classical test theory [CTT; in this study, it included discrete trend, exploratory factor analysis (EFA), correlation coefficient and Cronbach's *α* coefficient] and the item response theory (IRT) to perform the selection of scale items.^13^ In a variety of CTT methods, the standard deviation (SD) of the score of each item is used to measure its discrete trend; it is recommended to delete items with an SD < 1.^13^, ^14^ In the EFA, the principal component method was used to analyse each factor and perform maximum orthogonal rotation. Items with low factor loading (<0.4) or close to other factors in the EFA were excluded. The correlation coefficient of each item with its dimension was calculated, and in the present study, items yielding small correlation coefficients (<0.6) were deleted.^15^ Internal consistency was assessed using Cronbach's *α* coefficient and corrected item‐total correlation (CITC). If there is an item with CITC < 0.5 or a large increase in the value of Cronbach's *α* coefficient after the item is removed, it indicates that its existence has the effect of reducing the internal consistency of the dimension and should be removed.^16^

IRT, as the last method of item selection of this study, evaluated item performance by constructing the Bayesian Generalized Partial Credit Model (GPCM). Through parallel and rawpar functions, the dimension settings were consistently unidimensional, and then IRT was applied in 10 dimensions. The study used the marginal maximum likelihood estimation method for large samples, and the discrimination parameter (a) and the difficulty parameter ($b_{i}$) for each item were calculated using Multilog 7.03 software. The general requirement of a is >0.6, and in the present study, items for which a was <0.60 were excluded. The $b_{1}$, $b_{2}$, $b_{3}$ and $b_{4}$ parameters correspond to four levels of difficulty, where $b_{1}$ is the category threshold parameter between option 1 and option 2, and so on, and $b_{1}$ < $b_{2}$ < $b_{3}$ < $b_{4}$. The range of difficulty level parameters is generally −3 to +3.^17^

After applying the above‐described five tests, if an item passed three or more, it was retained. The items thus retained were combined with additional items identified by the majority of experts on an expert panel of investigators via a formal process described in detail below, to generate the final version of the scale.

### 2.4 Validation of the scale

Assessment of a scale generally includes reliability analysis, validity analysis and feasibility analysis. The validity analysis used in the current study involved content validity, dimensional correlation, construct validity and reactivity analysis.

### 2.5 Reliability analysis

Reliability refers to the consistency of measurement results. The most commonly used indicator of reliability is Cronbach's *α* coefficient. It is generally believed that Cronbach's *α* coefficient should be above 0.7.

### 2.6 Content validity

Content validity refers to whether the items can represent the measured content. In the present study, the content validity index (CVI) was used for quantitative analysis. If the CVI was higher than 80%, the item was retained.

### 2.7 Dimensional correlation

Dimensional correlation is the degree of correlation between the item and its own domain. When the correlation coefficient *r* is >.50, the dimensional correlation is considered acceptable.

### 2.8 Construct validity

The validity of a construct is an indicator of whether its domain can be evaluated via confirmatory factor analysis. LISREL 10.0 software was used to conduct these calculations in the present study. In accordance with the theoretical framework, a four‐factor model was tested. While there are many fitting indexes available for model evaluation, none of them can be used as a completely standardized test of the success of a model. Relatively reliable indicators include the non‐normed fit index (NNFI), the comparative fit index (CFI), the adjusted goodness‐of‐fit index (AGFI) and the approximate error root mean square (RMSEA). It is generally believed that an RMSEA value below 0.08 corresponds to a reasonably good fit (the smaller the better), and a value between 0.08 and 0.10 indicates a moderate degree of fitting. As with the RMSEA, the smaller the root mean square residual value, the better the fit. When the normative fit index, the NNFI, the CFI and the value‐added fit index are above 0.9, the fitting is considered reasonably good (the bigger the better),^18^ and when they are close to 0.90, the degree of fitting can be considered acceptable.

### 2.9 Response analysis

Response analysis refers to the ability of items to measure small changes in indicators. Whether the scale can determine changes in an indicator in the same group over time is often investigated, as is whether it can identify differences in a measured indicator between different groups. It reflects an ability to determine the characteristics of different populations. In the present study, the scale was required to be able to distinguish between the CRF group and the control group based on differences between their mean scores in each dimension (except the nonapplicable therapeutic dimension). The statistical method used was the two‐sample *t* test, and *p* <.05 was deemed to indicate that the scale was able to differentiate between the control group and the CRF group.

### 2.10 Feasibility analysis

Feasibility reflects the degree to which the scale is deemed acceptable by target respondents. Commonly used indicators include the completion rate and the completion time. The completion rate, also known as the response rate, refers to the percentage of questionnaires that are attempted by respondents and returned. It is generally required to be more than 85%. The scale completion time is usually intentionally restricted to 30 min or less, because a longer duration is not conducive to clinical application or the implementation of an investigation.

### 2.11 Data analysis software

Data analyses were conducted using SPSS 25.0, Multilog 7.03, LISREL 10.0 software and R 3.6.1.

## 3 RESULTS

### 3.1 Participant characteristics

Table 1 shows the characteristics of the 364 CRF patients and 125 control patients who completed the final scale. The sociodemographic characteristics of the two groups of participants are balanced and comparable.

**Table 1:** Demographic characteristics of the CRF group and the control group

Variables	Category	CRF group (*n* = 364)	Control group (*n* = 125)	$t/\chi^{2}$	*p*
Age		68.71 ± 12.27	66.54 ± 13.52	1.662	.097
Gender	Male	218 (59.9%)	66 (52.8%)	1.921	.166
	Female	146 (40.1%)	59 (47.2%)		
Marital status	Unmarried	15 (4.1%)	5 (4.0%)	2.022	.568
	Married	308 (84.6%)	100 (80%)		
	Widowed	36 (9.9%)	17 (13.6%)		
	Divorced	5 (1.4%)	3 (2.4%)		
Educational level	Elementary school and below	53 (14.6%)	22 (17.6%)	2.931	.402
	Junior high school	128 (35.1%)	47 (37.6%)		
	High school	96 (26.4%)	35 (28.0%)		
	College and above	87 (23.9%)	21 (16.8%)		
Occupation	Farmer	98 (26.9%)	37 (29.6%)	3.405	.638
	Worker	125 (34.3%)	40 (32.0%)		
	Staff	40 (11.0%)	13 (10.4%)		
	Professional skill worker	36 (9.9%)	9 (7.2%)		
	Manager	27 (7.4%)	7 (5.6%)		
	Others	38 (10.5%)	19 (15.2%)		

Abbreviation: CRF, chronic respiratory failure.

### 3.2 Item selection

The discrete trend is measured using the SD. The SD of each item is shown in Table 2. The recommended deletion (SD < 1) is COM1\SAT4. In factor analysis, Kaiser–Meyer–Olkin = 0.795, and Bartlett's spherical test yielded *p* < .001, indicating that the data were highly suitable for factor analysis. Based on the theoretical framework and the results of EFA, 10 factors were ultimately selected. Variance maximization orthogonal rotation was then applied, and items for which the factor loading on each factor was small (<0.4) or similar were removed (Table 2). The recommended deletions were SOM4, SOM12, SOM13, SOM14, FOC4, DLA1, ANX1, DEP2, DEP4, SC1, SC5 and FS1.

**Table 2:** Results of the item‐selection phase using CTT and IRT

Item	SD	Factor loading	Correlation coefficient	CITC	CAID	IRT					Outcome
						*α*	*b*_1_	*b*_2_	*b*_3_	*b*_4_	
SOM1	1.250	0.753	0.742	0.692	0.904	1.233	−1.271	0.227	−0.201	2.399	√
SOM2	1.270	0.707	0.734	0.682	0.904	1.139	−1.695	−0.466	−0.260	1.110	√
SOM3	1.150	0.455	0.651	0.595	0.907	0.730	−2.360	−1.115	−1.269	1.026	√
SOM4	1.150	0.437	0.614	0.553	0.908	0.667	−1.904	−1.697	−1.407	0.245	√
SOM5	1.440	0.718	0.824	0.782	0.899	2.183	−1.260	−0.351	−0.356	0.363	√
SOM6	1.450	0.735	0.776	0.724	0.902	1.629	−1.141	−0.381	−0.444	0.409	√
SOM7	1.380	0.746	0.760	0.708	0.903	1.386	−1.612	−0.605	−0.321	0.063	√
SOM8	1.350	0.744	0.751	0.699	0.903	1.476	−1.285	−0.776	−0.407	0.338	√
SOM9	1.380	0.684	0.703	0.642	0.905	0.791	−0.351	0.060	−0.238	1.960	√
SOM10	1.290	0.539	0.524	0.444	0.932	0.439	−2.090	−0.103	−0.580	1.800	X
SOM11	1.370	0.653	0.706	0.646	0.905	0.889	−1.465	−0.353	−0.194	0.656	√
SOM12	1.260	0.303	0.567	0.494	0.940	0.463	−1.016	−2.029	−1.318	0.327	X
SOM13	1.490	0.189	0.552	0.462	0.933	0.367	1.388	−1.358	−1.500	0.888	X
SOM14	1.370	0.289	0.659	0.592	0.907	0.685	−1.257	−0.337	−1.524	0.404	√
FOC1	1.330	0.732	0.839	0.680	0.727	1.865	−1.248	−0.748	−0.716	0.220	√
FOC2	1.280	0.591	0.755	0.552	0.701	0.905	−1.703	−0.581	−1.243	0.104	√
FOC3	1.260	0.699	0.832	0.681	0.732	1.879	−1.522	−0.712	−0.713	0.586	√
FOC4	1.380	0.390	0.627	0.340	0.816	0.325	0.487	−2.314	−1.501	−0.563	X
DLA1	1.400	0.077	0.430	0.014	0.812	1.826	−0.270	0.056	0.559	1.421	X
DLA2	1.460	0.714	0.714	0.449	0.652	‐0.049	−6.567	−6.374	9.962	−4.478	√
DLA3	1.510	0.880	0.861	0.692	0.753	0.053	13.041	10.155	−14.334	6.827	√
DLA4	1.460	0.861	0.824	0.633	0.710	1.009	−1.132	−0.125	−0.524	0.677	√
ANX1	1.310	0.437	0.751	0.633	0.875	1.058	−1.526	0.049	−0.963	0.946	√
ANX2	1.260	0.511	0.816	0.727	0.860	1.550	−1.318	−0.359	−0.432	1.236	√
ANX3	1.270	0.496	0.851	0.776	0.852	2.111	−1.342	−0.715	−0.183	0.806	√
ANX4	1.350	0.653	0.817	0.720	0.861	1.650	−1.001	−0.202	0.296	0.960	√
ANX5	1.370	0.696	0.746	0.620	0.878	0.903	−1.474	0.337	−0.342	0.938	√
ANX6	1.220	0.608	0.806	0.718	0.862	1.419	−2.132	−0.213	−0.876	0.637	√
DEP1	1.280	0.535	0.658	0.523	0.832	0.714	−1.520	−0.929	−0.922	0.473	√
DEP2	1.320	0.480	0.663	0.525	0.832	0.632	−1.539	−0.149	0.489	0.960	X
DEP3	1.340	0.513	0.723	0.600	0.821	0.922	−1.253	−0.400	−0.959	0.582	X
DEP4	1.220	0.550	0.670	0.546	0.829	0.801	−1.598	−1.303	−1.190	−0.679	X
DEP5	1.220	0.668	0.772	0.678	0.810	1.322	−2.042	−0.618	−1.124	−0.029	√
DEP6	1.390	0.603	0.746	0.624	0.817	1.028	−1.258	−0.155	−0.990	0.478	√
DEP7	1.320	0.655	0.790	0.691	0.806	1.690	−1.215	−0.765	−0.921	0.291	√
INF1	1.240	0.759	0.703	0.579	0.764	1.164	−1.213	−0.976	−1.170	−0.778	√
INF2	1.400	0.386	0.636	0.472	0.783	0.629	−0.041	−1.795	−0.477	−0.521	√
INF3	1.300	0.477	0.606	0.448	0.786	0.553	−0.561	−1.213	−1.937	−0.270	√
INF4	1.420	0.576	0.676	0.520	0.804	0.687	0.164	−1.590	−0.766	−0.465	X
INF5	1.300	0.611	0.718	0.591	0.761	1.120	−1.336	−0.846	−0.845	−0.606	√
INF6	1.250	0.621	0.677	0.544	0.770	1.010	−0.688	−1.995	−0.472	−1.236	√
INF7	1.390	0.712	0.700	0.556	0.767	0.881	−0.194	−0.862	−1.649	−0.366	√
SC1	1.360	0.339	0.307	0.060	0.855	0.037	−5.197	5.143	6.423	2.387	X
SC2	1.590	0.748	0.807	0.658	0.675	0.858	0.084	0.182	−0.299	0.320	√
SC3	1.620	0.796	0.823	0.679	0.666	1.687	0.029	−0.244	0.225	0.439	√
SC4	1.520	0.810	0.859	0.749	0.642	3.849	−0.610	−0.043	0.286	0.771	√
SC5	1.470	0.507	0.745	0.579	0.707	0.775	−0.755	−0.451	−0.284	−0.091	√
FS1	1.410	0.784	0.864	0.738	0.775	1.642	−1.341	−0.683	−0.547	−0.115	√
FS2	1.430	0.795	0.889	0.780	0.754	2.406	−1.308	−0.440	−0.409	0.297	√
FS3	1.520	0.708	0.856	0.707	0.792	1.569	−0.788	−0.438	−0.835	0.042	√
FS4	1.060	0.702	0.679	0.518	0.864	0.843	−2.393	−2.085	−1.034	−1.216	√
COM1	0.950	0.706	0.761	0.541	0.686	1.515	−2.696	−2.164	−0.940	−0.324	√
COM2	1.360	0.544	0.835	0.530	0.686	1.055	−1.155	−0.900	−0.721	0.135	√
COM3	1.180	0.576	0.776	0.486	0.726	0.776	−2.471	−1.478	−0.621	−0.066	√
SAT1	1.270	0.649	0.703	0.566	0.761	0.603	−2.593	−1.299	0.089	−0.637	√
SAT2	1.230	0.540	0.594	0.432	0.883	0.481	−1.761	−2.616	−0.121	−1.312	X
SAT3	1.310	0.722	0.557	0.372	0.895	0.278	0.900	−4.714	−0.368	−2.531	X
SAT4	0.860	0.545	0.641	0.541	0.770	0.476	−0.969	−3.463	−1.744	−2.232	√
SAT5	1.200	0.636	0.746	0.631	0.750	1.618	−1.614	−1.568	−0.363	−0.400	√
SAT6	1.090	0.699	0.675	0.553	0.764	1.294	−1.679	−2.037	−0.634	−0.822	√
SAT7	1.100	0.753	0.633	0.498	0.772	1.032	−2.138	−1.512	−1.024	−0.560	√
SAT8	1.040	0.683	0.612	0.481	0.775	0.779	−2.074	−2.664	−0.654	−1.030	√

Abbreviations: ANX, anxiety; CAID, Cronbach's *α* if item deleted; CITC, corrected item‐total correlation; COM, compliance; CTT, classical test theory; DEP, depression; DLA, daily living activity; FOC, function of cognitive; FS, family support; INF, inferiority; IRT, item response theory; SAT, satisfaction; SC, social contacts; SD, standard deviation; SOM, somatization; ‘√’ in the table is the selected item ‘X’ to delete the item.

In accordance with the predetermined theoretical structure, the correlation coefficient of each item with its dimension and other dimensions was calculated and items with small correlation coefficients (<0.6) were deleted (Table 2). The suggested deletions were SOM10, SOM12, SOM13, DAL1, SC1, SAT2 and SAT3. The Cronbach's *α* coefficient for each dimension of the scale is shown in Table 2. The suggested deletions were SOM10, SOM12, SOM13, FOC4, DLA1, INF4, SC1, SC5 and FS1.

**Figure 3:**
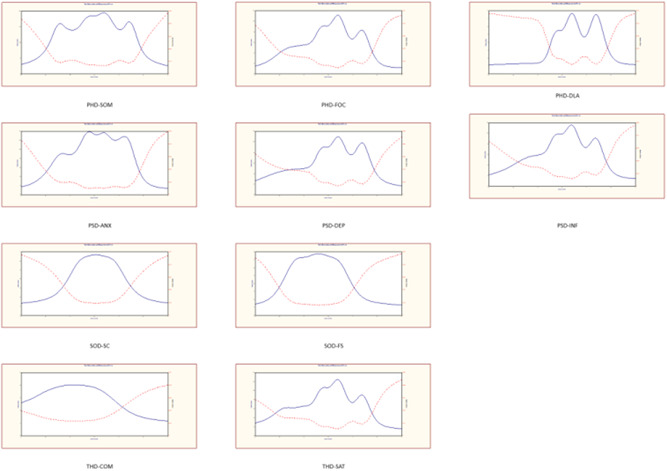
Test information and measurement error for each dimension. ANX, anxiety; COM, compliance; DEP, depression; DLA, daily living activity; FOC, function of cognitive; FS, family support; INF, inferiority; PHD, physiological domain; SAT, satisfaction; SC, social contacts; SOD, social domain; SOM, somatization; THD, therapeutic domain.

**Figure 4:**
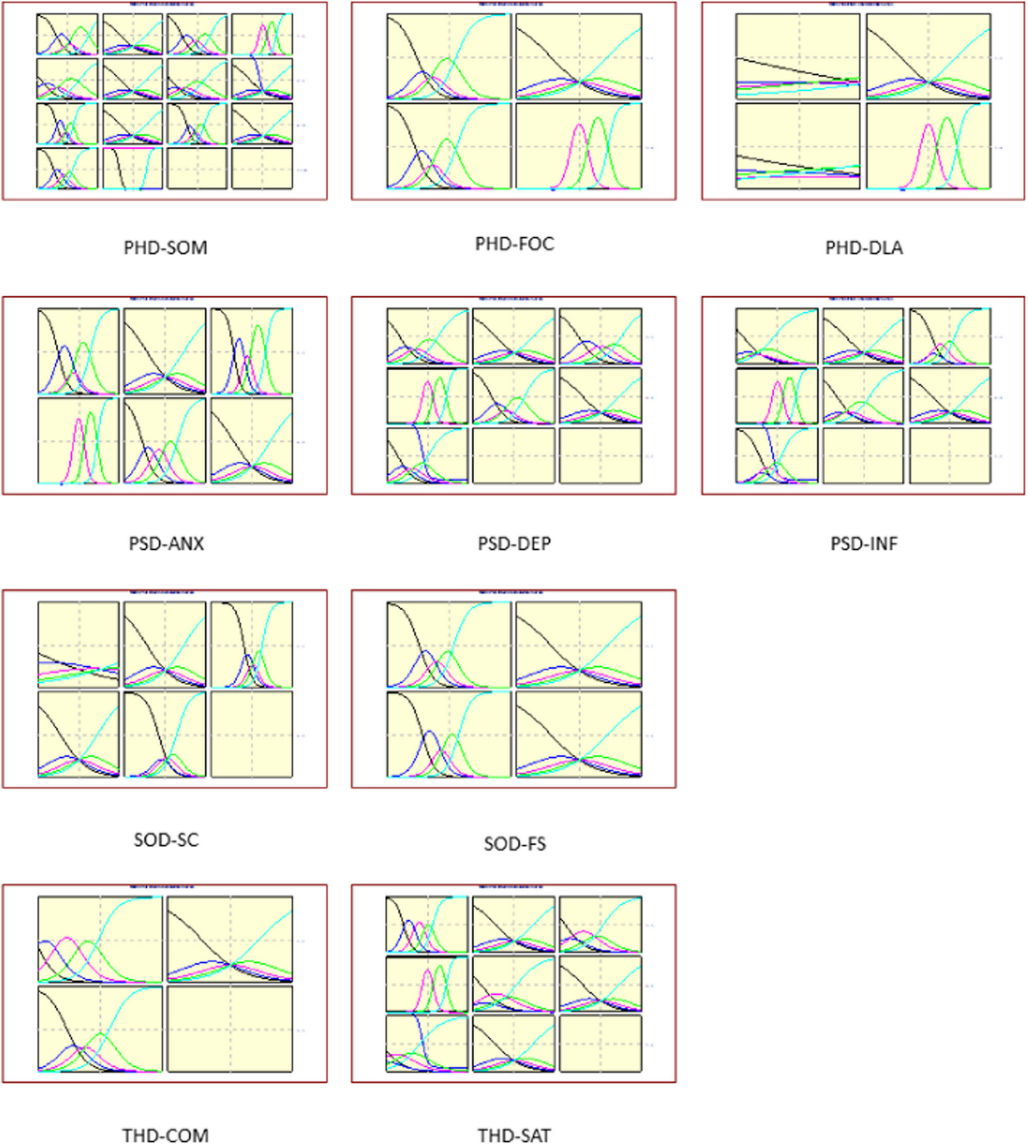
Matrix plot of item characteristic curves:  (1) black, (2) blue, (3) magenta, (4) green and (5) cyan

The results of the unidimensional test are presented in Appendix B. When only one factor is extracted (the eigenvalue of the second factor is less than 1) or the eigenvalue of the first factor is greater than two times the second factor, the dimension is considered to be unidimensional. Based on IRT, considering the estimated values of $a_{i}$, the suggested deletions were DLA1, DLA3 and SC1 (Table 2). Figure 3 shows the total amount of information and measurement error provided by each dimension. Figure 4 shows a matrix diagram of the item characteristic curve (ICC) of each item. Ideally, the first and fifth curves of the ICC change monotonically, and the second, third and fourth curves are normally distributed. As shown in Figure 4, the characteristic curve distributions of most items were ideal.

Items fulfilled the requirements of at least three of the five assessment methods applied and were thus retained are shown in Appendix A. The final scale included a total of 50 items: 17 in the physiological domain, 16 in the psychological domain, 8 in the social domain and 9 in the therapeutic domain.

### 3.3 Evaluation of the scale

#### 3.3.1 Reliability analysis

The calculation of Cronbach's *α* coefficient was conducted in the four domains internally. Cronbach's *α* coefficients were 0.870 in the physiological domain, 0.877 in the psychological domain, 0.807 in the social domain, 0.712 in the therapeutic domain and for the entire scale, it was 0.905. Overall, the scale showed excellent reliability.

#### 3.3.2 Content validity

The CVI of all items was higher than 80%, indicating good content validity.

#### 3.3.3 Dimensional correlation

In the final investigation, there were strong correlations between the items and their domains. The correlation coefficients of all 17 items in the physiological function domain were above 0.50, those of 13 of the 16 items in the psychological function domain were above 0.50, those of all 8 items in the social support domain were above 0.50 and those of 7 of the 9 items in the therapeutic domain were above 0.50.

#### 3.3.4 Construct validity

There was a four‐factor structure in confirmatory factor analysis; in the physiological domain, 17 items corresponded to three latent variables, in the psychological domain, 16 items corresponded to three latent variables, in the social domain, 8 items corresponded to two latent variables and in the therapeutic domain, 9 items corresponded to two latent variables (Table 3). Fitting indexes supported the model, and the scale showed high structural validity (Table 4). The CFA construct frameworks and standard estimates in four domains are shown in Appendix C.

**Table 3:** Confirmatory factor analysis results for each dimension corresponding to each item

Dimension	Item	Nonstandard factor loading	Standard error	Standard factor loading	t
SOM	PHD1	0.71	0.07	0.62	12.47
	PHD2	0.81	0.05	0.72	15.35
	PHD3	0.73	0.06	0.65	13.31
	PHD4	0.71	0.06	0.64	13.06
	PHD5	0.91	0.06	0.74	15.73
	PHD6	0.93	0.07	0.72	15.23
	PHD7	0.77	0.08	0.60	12.10
	PHD8	0.81	0.08	0.63	12.88
	PHD9	0.60	0.11	0.45	8.53
	PHD10	0.77	0.10	0.58	10.92
	PHD11	0.63	0.12	0.45	8.65
FOC	PHD12	1.03	0.11	0.69	13.27
	PHD13	0.99	0.09	0.72	14.22
	PHD14	0.94	0.09	0.72	14.00
DLA	PHD15	0.70	0.11	0.50	9.41
	PHD16	1.25	0.12	0.92	17.11
	PHD17	1.02	0.10	0.75	14.04
ANX	PSD1	0.74	0.06	0.60	11.84
	PSD2	0.88	0.06	0.72	15.08
	PSD3	0.97	0.06	0.75	16.02
	PSD4	1.02	0.06	0.78	16.85
	PSD5	0.96	0.06	0.74	15.60
	PSD6	0.87	0.06	0.70	14.42
DEP	PSD7	0.97	0.08	0.65	12.20
	PSD8	1.09	0.08	0.71	13.30
	PSD9	0.65	0.07	0.53	7.50
	PSD10	0.59	0.08	0.55	7.89
INF	PSD11	0.86	0.08	0.58	11.28
	PSD12	0.59	0.08	0.52	7.70
	PSD13	0.77	0.08	0.59	9.29
	PSD14	1.15	0.07	0.77	16.15
	PSD15	1.01	0.06	0.76	15.91
	PSD16	0.94	0.07	0.69	13.94
SC	SOD1	0.93	0.07	0.64	11.92
	SOD2	1.27	0.06	0.90	19.98
	SOD3	1.17	0.06	0.85	18.36
	SOD4	0.70	0.08	0.52	3.91
FS	SOD5	0.52	0.08	0.48	3.97
	SOD6	1.15	0.07	0.93	17.46
	SOD7	1.03	0.07	0.83	15.59
	SOD8	0.58	0.07	0.50	2.08
COM	THD1	0.55	0.06	0.53	2.57
	THD2	0.58	0.06	0.51	9.07
	THD3	0.95	0.07	0.89	13.92
SAT	THD4	0.97	0.06	0.83	16.49
	THD5	0.65	0.06	0.61	11.47
	THD6	0.61	0.06	0.57	10.55
	THD7	0.62	0.06	0.52	3.83
	THD8	0.77	0.06	0.66	2.80
	THD9	0.68	0.06	0.56	2.84

Abbreviations: ANX, anxiety; COM, compliance; DEP, depression; DLA, daily living activity; FOC, function of cognitive; FS, family support; INF, inferiority; PHD, physiological domain; PSD, psychological domain; SAT, satisfaction; SC, social contacts; SOD, social domain; SOM, somatization; THD, therapeutic domain.

**Table 4:** Goodness‐of‐fit statistics of the CRF‐PROM

	RMSEA	RMR	NFI	NNFI	CFI	IFI
PHD	0.078	0.097	0.86	0.88	0.90	0.90
PSD	0.089	0.073	0.90	0.90	0.91	0.91
SOD	0.099	0.087	0.88	0.84	0.89	0.90
THD	0.095	0.090	0.80	0.79	0.82	0.82
Total	0.086	0.078	0.82	0.85	0.86	0.86

Abbreviations: CFI, comparative fit index; CRF, chronic respiratory failure; IFI, incremental fit index; NFI, normative fit index; NNFI, non‐normed fit index; PHD, physiological domain; PROM, patient‐reported outcome measure; PSD, psychological domain; RMR, root mean square residual; RMSEA, root mean square error of approximation; SOD, social domain; THD, therapeutic domain.

#### 3.3.5 Response analysis

In the response analysis, the mean scores derived from the control group and the CRF group were statistically significantly different in every dimension, indicating that the scale has the ability to distinguish between people with different qualities of life (Table 5).

**Table 5:** Comparison between the CRF group and the control group

Dimension	CRF group	Control group	t	*p*
SOM	35.59 ± 8.96	49.00 ± 5.82	13.973	<.001
FOC	9.39 ± 3.40	13.34 ± 2.11	−14.182	<.001
DLA	8.18 ± 3.36	13.55 ± 2.17	−19.007	<.001
ANX	19.95 ± 5.85	26.33 ± 3.68	−13.184	<.001
DEP	12.23 ± 3.99	16.91 ± 3.73	−10.832	<.001
INF	19.84 ± 6.06	26.56 ± 3.72	−13.630	<.001
SC	10.32 ± 4.95	13.19 ± 4.84	−5.093	<.001
FS	14.04 ± 3.61	16.70 ± 3.59	−6.458	<.001

Abbreviations: ANX, anxiety; CRF, chronic respiratory failure; DLA, daily living activity; DEP, depression; FOC, function of cognitive; FS, family support; INF, inferiority; SC, social contacts; SOM, somatization.

#### 3.3.6 Feasibility analysis

In the small sample investigation, we sent initial PROMs out to 120 patients with CRF. One hundred and twenty PROMs were sent out and 112 were returned, representing a response rate of 93.3%. In the 112 returned PROMs, 102 (91.1%) were valid. In the large sample investigation, 498 of the 510 questionnaires sent out were returned, corresponding to a response rate of 97.6%. Of the 498 questionnaires returned, 489 were valid (364 from patients with respiratory failure and 125 from control patients), corresponding to a validity rate of 98.2%. With regard to completion time, the average time spent performing scale data collection was approximately 15 min. The above results indicate that the scale is feasible.

## 4 DISCUSSION

In the present study, a CRF‐PROM to evaluate outcomes in patients with CRF was developed and validated. To the best of our knowledge, it is the first CRF‐PROM specifically developed and validated for posthospital management and clinical trials of medical products. It includes physical, psychological, social and therapeutic domains, and a total of 50 items. The average time spent performing scale data collection was approximately 15 min.

The scale addresses the current lack of a quality‐of‐life assessment tool specifically designed for use by patients with CRF. CRF occurs during the end stage of many respiratory diseases, particularly COPD.^2^ Researchers often ignore the difference between COPD and CRF. There are currently many quality‐of‐life scales available for use in COPD patients and they have been widely used in clinical research,^19^, ^20^ but no quality‐of‐life scale has been specifically developed for use in patients with CRF. Most of the patients involved in the present study had primary diseases such as pneumonia, emphysema, pleural effusion or asthma with associated COPD (52.2%), which further deteriorated into CRF. Another major cause of CRF in the patients in the present study was pulmonary heart disease (30.2%), and some had other causes of respiratory failure such as pulmonary hypertension, tuberculosis or silicosis. Various types of patients were included in the study, so the applicability of the scale is evidently broad, and it is suitable for use in patients with CRF caused by any primary disease.

Quality‐of‐life research conducted in China has historically involved the use of questionnaires that have been translated from another language. As a result, some of the items have been inconsistent with some habits typical of Chinese people, particularly habits pertaining to inherently personal practices, or questions about habits that many Chinese people would consider to be sensitive areas of inquiry—resulting in potential bias. The scale developed in the current study via discussion with specialists and interviews with CRF patients addresses this applicability problem with regard to patients in China.

Notably, via in‐depth interviews with 10 patients, we identified some concerns that have not been previously reported. For example, some patients reported feeling forgetful after they entered a disease stage involving CRF. To date, there is not enough evidence to definitively show that this is caused by the CRF, but we retained relevant items in the CRF‐PROM, and intend to further explore this potential phenomenon in future studies. Importantly, at the end of the CRF‐PROM, we have included 'free‐response' items to ensure that patients will have the opportunity to allude to any effects they have experienced that are not explicitly referred to in the scale.

Changes in social support and social support utilization after the onset of illness are often overlooked in the development of HRQOL scales. Wada et al.^21^ reported that reduction in the social support received by patients with CRF had the potential to cause mood disorders such as depression. Therefore, in the item development phase, we paid due attention to changes in social support, and awarded it the same status as the therapeutic domain. The patient's tolerance of therapeutic costs, satisfaction with doctors and other factors that are easily overlooked have also been incorporated into the CRF‐PROM.

The item selection phase involved analyses based on both CTT and IRT. In the development of existing scales, researchers have generally used CTT to conduct item selection (e.g., EFA, Cronbach's *α* coefficient).^22^, ^23^ IRT‐based analysis has been used more frequently in the construction of scales for measuring subjective attributes than CTT‐based analysis. IRT‐based analysis also facilitates more accurate examination of the features of each scale item than CTT‐based analysis. CTT statistics are associated with certain disadvantages, whereas methods based on IRT offer several advantages pertaining to the refinement of items, thus improving on analyses conducted using CTT alone.^24^

CRF is a long‐term disease, so posthospital management plays an important role in a patient's prognosis.^25^ Because of a relative lack of medical knowledge, however, family members often do not know how to systematically assess changes in a patient's status. In this context, it is reasonable to expect that the CRF‐PROM will assist caregivers.

The National Center for Complementary and Alternative Medicine emphasizes that the effect of an intervention or treatment must be confirmed by a recognized endpoint indicator.^26^ To complement established indicators (e.g., minute ventilation), the CRF‐PROM can be used as an endpoint indicator in clinical research that is more focused on the patient's subjective feelings. It is also suitable for follow‐up examinations, and it is more economical and easier to implement than some established indicators.

Foreseeably, the CRF‐PROM has several potential advantages in the clinical trial phase of medical product development. From a researcher's perspective, the scale may capture the patient's experience and treatment benefit or risk, assist in determining which patients with CRF benefit meaningfully from treatment and facilitate between‐trial comparisons.^6^ From a pharmaceutical company's perspective, such an instrument may increase the efficiency of discussions with the FDA during the medical product development process and provide valuable information derived directly from patients for use in drawing conclusions about treatment effects during the process of consideration for medical product approval.^6^ From a regulatory perspective, the CRF‐PROM may provide a standardized method for assessing the effectiveness of treatment of basic symptoms, such that claims can be supported with CRF‐PROM‐based evidence in medical product clinical trials.^27^ While developed countries have used a variety of quality‐of‐life assessment tools widely in clinical trials of new medical products, in developing countries such as China, the practice is relatively uncommon. Considering the above‐described advantages, the CRF‐PROM will help to change this situation. Lastly, given its demonstrated validity and reliability, the CRF‐PROM is appropriate for broad‐scale use in the posthospital management of patients with CRF as well as the development of medical products.

There were some inevitable problems associated with the use of the CRF‐PROM in the current study. One was that because most of our investigations were conducted in the inpatient department of the hospital, and during hospitalization patients with CRF usually have severe symptoms, some of the patients were unable to complete the scale unassisted. In such cases, the investigators assisted the patients in completing the scale. The proportion of patients who were not able to complete the CRF‐PROM unassisted was higher than expected and it may have resulted in a degree of concealment in some domains, particularly in the psychological domain. Second, the patients in this study came from provincial hospitals, municipal hospitals and community hospitals, and their incomes varied substantially. Thus, with regard to use in different types of hospitals and in patients with diverse economic status, the applicability of the scale was good. Because of funding and personnel limitations, the representativeness of the sample was still not ideal, however, in that it was derived from a single province. We intend to conduct investigations in additional provinces in the future, to enhance the representativeness of the cumulative sample and the reliability of the results.

## 5 CONCLUSIONS

The results of the present study suggest that the CRF‐PROM is effective and reliable. It can be widely used in the posthospital management of patients, studies investigating CRF and clinical trials of new medical products. The CRF‐PROM addresses the current lack of a scale specifically designed for use in CRF patients, and it may also prove useful in the development of additional disease‐specific scales. Notably, however, the broader applicability of the scale needs to be assessed via administration in more provinces of China, and its wider generalizability in different races also remains to be assessed. It may require additional ongoing modifications.

## CONFLICT OF INTERESTS

The authors declare that there are no conflict of interests.

## ETHICS APPROVAL AND CONSENT TO PARTICIPATE

The study and the CRF‐PROM were reviewed and approved by the Medical Ethics Committee of Shanxi Medical University, China (No. 2018LL128), and written informed consent was obtained from all participants.

## AUTHOR CONTRIBUTIONS

All authors participated in the design of the study. Hangzhi He participated in drafting the manuscript and revised the article. Hao Li participated in researcher coordination, data analysis and drafting the manuscript. Xianhua Zeng was primarily responsible for data collection and analysis. Yanbo Zhang and Hui Zhao conceptualized the study, supervised data analysis and oversaw the generation of the original manuscript. All authors have read and approved the final manuscript.

## Figures and Tables

**Figure C1:**
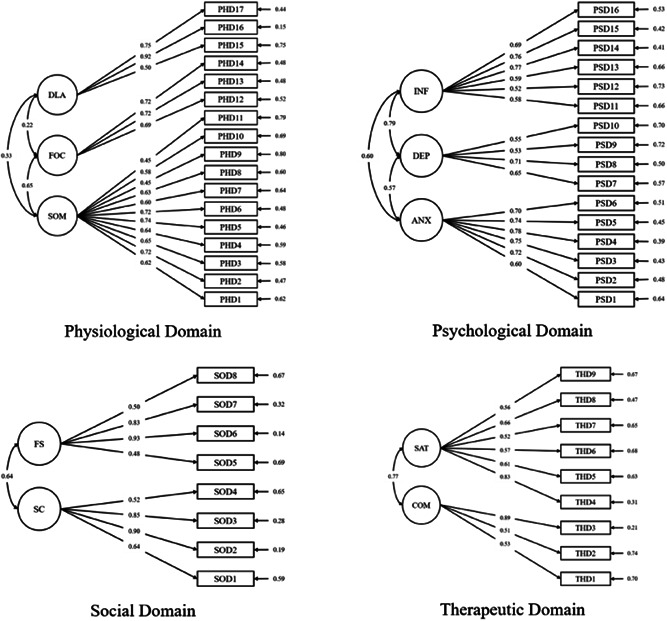
CFA construct frameworks and standard estimates in four domains of the CRF‐PROM. ANX, anxiety; CFA, confirmatory factor analysis; COM, compliance; CRF, chronic respiratory failure; DEP, depression; DLA, daily living activity; FOC, function of cognitive; FS, family support; INF, inferiority; PHD, physiological domain; PROM, patient‐reported outcome measure; PSD, psychological domain; SAT, satisfaction; SC, social contacts; SOD, social domain; SOM, somatization; THD, therapeutic domain

## Data Availability

The data that support the findings of this study are available on request from the corresponding authors. The data are not publicly available due to privacy or ethical restrictions.

## APPENDIX A

**Table A1:** Final scale

Item
PHD1 Do you experience shortness of breath?
PHD2 Do you often feel chest tightness?
PHD3 Do you often feel dizzy?
PHD4 Do you get headaches often?
PHD5 Do you experience difficulty breathing when you eat?
PHD6 Do you experience difficulty breathing when you bend over?
PHD7 Do you experience difficulty breathing when you stand up from sitting on a chair or couch?
PHD8 Do you experience difficulty breathing when you speak?
PHD9 Do you cough often?
PHD10 Do you experience shortness of breath even in the absence of any physical activity?
PHD11 Have you ever woken up at night due to shortness of breath?
PHD12 Are you more likely to forget people's names than you ever have been before?
PHD13 Are you very forgetful?
PHD14 Do you often forget what you wanted to say after you begin talking?
PHD15 Can you go for walks alone to buy general goods such as groceries?
PHD16 Can you do general housework such as cooking?
PHD17 Do you experience difficulty breathing during daily activities such as washing or dressing?
PSD1 Are you prone to feelings of nervousness, irritability, or tension that you attribute to your ongoing illness?
PSD2 Do you often feel upset because of illness?
PSD3 Do you often feel nervous?
PSD4 Do you often feel sleepy during the day, and often wake up during the night?
PSD5 Are you worried that your illness is getting worse?
PSD6 Are you often upset by being alone?
PSD7 Do you often feel lonely for reasons that relate to illness?
PSD8 Are you uninterested in others or things around you?
PSD9 Do you feel that you are a burden on your family because of your illness?
PSD10 Are you often in a bad mood even if the atmosphere around you is very cheerful?
PSD11 Are you unwilling to socialize with others?
PSD12 Do you often feel sad?
PSD13 Are you afraid of your illness?
PSD14 Are you afraid of empty rooms or streets and need to be accompanied?
PSD15 Do you often feel uncomfortable when you are in an unfamiliar crowd?
PSD16 Do you often have no confidence in yourself?
SOD1 Have you given up some of your original hobbies due to illness (such as dancing or playing chess)?
SOD2 Does your illness affect your family life?
SOD3 Have you reduced your contact with friends and acquaintances because of your illness?
SOD4 Do you avoid particular social or family activities due to illness?
SOD5 Does your family take the initiative to address your needs in daily life?
SOD6 Are friends and relatives concerned about your condition?
SOD7 Do members of your family often remind you to take medicine?
SOD8 Do you think your family understands and tolerates your physical and emotional changes since your illness?
THD1 Do you follow your doctor's instructions with regard to taking correct doses of medicine regularly?
THD2 Do you follow your doctor's instructions with regard to abstaining from bad habits?
THD3 Do you follow your doctor's instructions with regard to visiting your medical institution regularly?
THD4 Do your current treatments effectively alleviate your symptoms?
THD5 Is your doctor friendly towards you while administering treatment?
THD6 Do you think all the things that your doctor asks you to do are necessary?
THD7 Are you willing to continue your current treatment plan?
THD8 Has your general confidence improved since you began receiving treatment?
THD9 Do you have a better understanding of your disease than you did before you began receiving treatment?

Abbreviations: PHD, physiological domain; PSD, psychological domain; SOD, social domain; THD, therapeutic domain.

## APPENDIX B

**Table B1:** Unidimensional test

Factor	Initial eigenvalues			Extraction sums of squared loadings		
	Eigenvalue	% of Variance	Cumulative %	Eigenvalue	% of Variance	Cumulative %
SOM						
1	6.541	46.719	46.719	6.541	46.719	46.719
2	1.578	11.268	57.987	1.578	11.268	57.987
3	1.046	7.474	65.461	1.046	7.474	65.461
4	0.946	6.755	72.216			
5	0.632	4.517	76.733			
6	0.597	4.262	80.995			
7	0.504	3.601	84.596			
8	0.448	3.199	87.795			
9	0.413	2.947	90.742			
10	0.340	2.430	93.172			
11	0.326	2.329	95.501			
12	0.260	1.857	97.358			
13	0.207	1.477	98.835			
14	0.163	1.165	100.000			
FOC						
1	2.344	58.599	58.599	2.344	58.599	58.599
2	0.847	21.168	79.767			
3	0.492	12.288	92.055			
4	0.318	7.945	100.000			
DLA						
1	1.585	39.618	39.618	1.585	39.618	39.618
2	1.449	36.220	75.837	1.449	36.220	75.837
3	0.545	13.632	89.470			
4	0.421	10.530	100.000			
DLA‐EXCEPT DLA1						
1	1.468	48.943	48.943	1.468	48.943	48.943
2	0.987	32.888	81.831			
3	0.545	18.169	100.000			
ANX						
1	3.839	63.976	63.976	3.839	63.976	63.976
2	0.804	13.403	77.379			
3	0.421	7.011	84.390			
4	0.379	6.324	90.714			
5	0.306	5.094	95.808			
6	0.252	4.192	100.000			
DEP						
1	3.595	51.364	51.364	3.595	51.364	51.364
2	0.810	11.567	62.931			
3	0.756	10.795	73.726			
4	0.607	8.67	82.397			
5	0.534	7.635	90.032			
6	0.434	6.205	96.236			
7	0.263	3.764	100.000			
INF						
1	3.219	45.983	45.983	3.219	45.983	45.983
2	1.132	16.169	62.152	1.132	16.169	62.152
3	0.731	10.437	72.589			
4	0.616	8.805	81.394			
5	0.510	7.279	88.673			
6	0.450	6.428	95.102			
7	0.343	4.898	100.000			
SC						
1	2.798	55.960	55.960	2.798	55.960	55.960
2	1.005	20.090	76.050	1.005	20.090	76.050
3	0.544	10.875	86.925			
4	0.435	8.707	95.633			
5	0.218	4.367	100.000			
FS						
1	2.697	67.436	67.436	2.697	67.436	67.436
2	0.664	16.611	84.047			
3	0.344	8.604	92.651			
4	0.294	7.349	100.000			
COM						
1	1.819	60.640	60.640	1.819	60.640	60.640
2	0.635	21.167	81.807			
3	0.546	18.193	100.000			
SAT						
1	2.982	37.276	37.276	2.982	37.276	37.276
2	1.257	15.713	52.988	1.257	15.713	52.988
3	0.886	11.078	64.066			
4	0.845	10.559	74.625			
5	0.702	8.781	83.406			
6	0.536	6.695	90.101			
7	0.419	5.237	95.338			
8	0.373	4.662	100.000			

Abbreviations: ANX, anxiety; COM, compliance; DEP, depression; DLA, daily living activity; FOC, function of cognitive; FS, family support; INF, inferiority; SAT, satisfaction; SC, social contacts; SOM, somatization.
